# Influence of Coating Material Thickness on the Attraction Force of Dental Magnetic Attachment: An In Vitro Study

**DOI:** 10.1155/2024/3863278

**Published:** 2024-05-16

**Authors:** An-Nissa Kusumadewi, Mikyal Delvi Luth Dewi, Lisda Damayanti, Rukiah Rukiah, Risdiana Risdiana

**Affiliations:** ^1^Department of Prosthodontics, Faculty of Dentistry, Universitas Padjadjaran, Jl. Raya Bandung-Sumedang Km 21, Jatinangor, Sumedang 45363, West Java, Indonesia; ^2^Faculty of Dentistry, Universitas Padjadjaran, Jl. Raya Bandung-Sumedang Km 21, Jatinangor, Sumedang 45363, West Java, Indonesia; ^3^Department of Chemistry, Faculty of Mathematics and Natural Sciences, Universitas Padjadjaran, Jl. Raya Bandung-Sumedang Km 21 Jatinangor, Sumedang 45363, West Java, Indonesia; ^4^Department of Physics, Faculty of Mathematics and Natural Sciences, Universitas Padjadjaran, Jl. Raya Bandung-Sumedang Km 21, Jatinangor, Sumedang 45363, West Java, Indonesia

## Abstract

**Objectives:**

This study aimed to determine the influence of coating material thickness on the attraction force of dental magnetic attachment (DMA).

**Materials and Methods:**

An in vitro experimental design was implemented using DMA as samples coated with different material types including polytetrafluoroethylene (PTFE) and glass ionomer varnish. DMA consists of keeper and assembly. The coating material was applied to DMA in two ways, on the assembly only and both the keeper and assembly. The thickness of each coated DMA was measured with a digital micrometer, and analysis was subsequently conducted with a universal testing machine to evaluate potential alterations in magnetic attraction force. Comparison was made between the attraction force of both the coated and uncoated DMA serving as a control specimen.

**Result:**

The thickness of the coating material applied to both the keeper and assembly was 25 *μ*m, while PTFE and varnish coating on the assembly alone was estimated as 12 and 10 *μ*m, respectively. The magnetic attraction force of the uncoated DMA was 613.63 gf. Following coating, the magnetic attraction force decreased by 34.02–79.45 gf. The ANOVA test indicated that the decrease in magnetic attraction observed across both types of coating material and technique did not show significant differences.

**Conclusion:**

The thickness and type of coating material had no significant effect on magnetic attraction.

## 1. Introduction

Dental magnetic attachment (DMA) is a type of prosthetic material commonly used in overdentures. Furthermore, it has a simple attachment procedure and self-seating capabilities, leading to being suitable for elderly patients with reduced motoric skills. The disadvantages of DMA include the possession of relatively low retentive force among other attachment types and susceptibility to corrosion, necessitating magnet replacement [1]. Previous studies showed the tendency of magnets to experience corrosion, which would trigger attraction force decline [2–5]. The production of overdentures with magnetic attachment is a time-consuming and costly process. DMA replacement in patients due to corrosion is inefficient. Despite coating the DMA with stainless steel, the risk of corrosion persists [3, 4, 6], suggesting the need for extra protection because the attachment is often placed in a corrosive oral environment.

Polytetrafluoroethylene (PTFE) is widely applied as a coating material for medical devices due to being chemically inert, physiologically stable, nontoxic, and nonflammable under normal conditions, without becoming metabolized [7]. PTFE is also used in coating neodymium iron boron (NdFeB) magnets to provide superior maintenance of flux density and other magnetic properties compared to uncoated NdFeB [8, 9].

Varnish prominently serves as a coating material for preventing glass ionomer cement from moisture contamination and dehydration during initial hardening [10, 11]. Tyagi et al. [12] reported the efficacy of varnish in protecting the surface of glass ionomer cement when exposed to nitric acid. The lack of sufficient literature on the exploration of additional protection for dental magnets with stainless steel casings using PTFE or varnish has stimulated the need to evaluate the optimal thickness of both materials. Therefore, this study aimed to determine the influence of coating material thickness on the attraction force of DMA.

## 2. Materials and Methods

**Figure 1:**
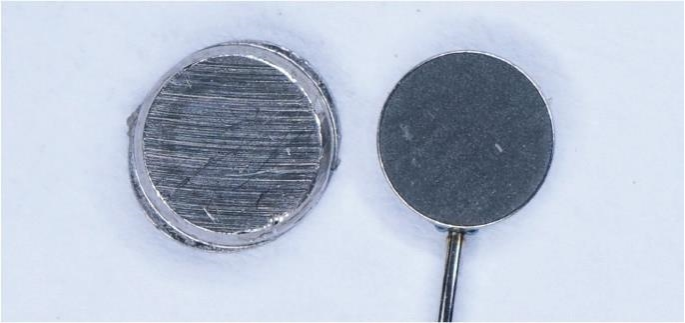
Magfit DX 600, comprising assembly and keeper component.

The samples used in this study were Magfit DX 600 (Aichi Steel, Japan), comprising assembly and keeper components (Figure 1). All were further categorized into five groups, including (1) PTFE coating on the assembly, (2) PTFE coating on the assembly and keeper, (3) varnish coating on the assembly, (4) varnish coating on the assembly and keeper, and (5) uncoated components as the control. Each group consisted of three samples, and this number was calculated based on the resource equation formula to determine the minimum sample size. The coating technique of PTFE material was modified from a previous study [8]. PTFE liquid was applied to the contact surface of the assembly/keeper using a microbrush, followed by gentle rubbing with a soft cloth. This procedure was repeated until the surface became evenly covered with the coating material. Varnish coating was performed by applying one layer of the liquid onto the contact surface of the assembly/keeper using a microbrush and allowing it to dry.

**Figure 2:**
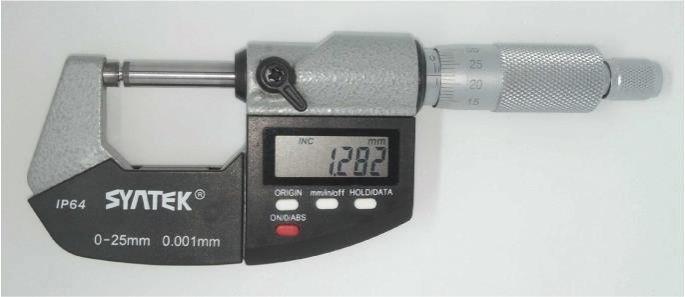
The thickness measurement.

Coated DMA groups were analyzed using a scanning electron microscope (SEM; Hitachi SU3500, Japan) to ensure uniform coverage on the entire assembly/keeper contact surface, while the control group was analyzed with SEM for comparison. The thickness of the entire surface area of the assembly and keeper in all groups was measured using a digital micrometer (Syntex, China), as shown in Figure 2. This measurement was conducted before and after coating with PTFE or varnish. The thickness of the coating material is obtained by calculating the difference in thickness between the coated DMA and uncoated DMA. The average thickness value for each group was determined based on the total measurement of samples.

The subsequent step included the preparation of DMA for attraction force measurement. In this context, the assembly and keeper in all groups were attached to aluminum rods using cyanoacrylic adhesive and then mounted onto a universal testing machine (UTM; Llyod LRX-Plus 5 kN, Ametek Inc., United Kingdom). The keeper was positioned on the lower UTM arm and the assembly was placed on the upper arm, each with a length of 1 cm [4]. The tensile test was conducted at a cross-head speed of 50 mm/min, and the maximum load value was taken [3, 13–16].

The attraction force in each sample was collected, and the statistical analysis was perfomed with MegaStat software V.10.4 release 3.2.4 Mac (McGraw Hill, USA). The mean and the standard deviation of each group were calculated. All the data were subjected to one-way analysis of variance (ANOVA) at a 95% level of confidence (*α* = 0.05).

## 3. Result

**Figure 3:**
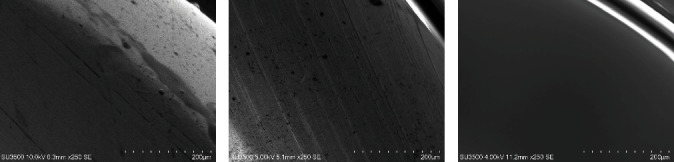
SEM analysis results for assembly surfaces: (a) after PTFE coated, (b) uncoated, and (c) after varnish coated.

The results of SEM analysis conducted in this study are presented at 250x magnification in Figure 3. These showed that the PTFE-coated assembly surface (Figure 3(a)) was different from the uncoated surface (Figure 3(b)) as well as the varnish-coated assembly surface (Figure 3(c)).

The average of magnetic attraction force of the uncoated DMA was 613.63 gf. The magnetic attraction force after coating decreased by 34.02–79.45 gf compared to the uncoated DM, as shown in Table 1. Data subjected to the ANOVA test (Table 2) showed a *p*-value of 0.0572 (*p*  > 0.05), indicating that no significant difference was found in magnetic attraction force across all groups.

## 4. Discussion

According to the results, the addition of coating material on the assembly/keeper contact surface was found to reduce the magnetic attraction force. The reduction of the magnetic attraction force was due to PTFE and varnish being nonferromagnetic materials. The study conducted by Ahmad et al. [8] showed that a thicker coating material would provide a greater barrier to the magnetic field. Ahmad et al. coated some groups of NdFeB magnets with one layer of PTFE and others with two layers of parylene. Consequently, the parylene group lost more magnetic flux density than the PTFE group [8]. In their study, the two layers of parylene produced a thickness of 20 *μ*m. This was consistent with the present study, where the thickness of the coating material for both the keeper and assembly was 25 *μ*m. Additionally, PTFE and varnish coating on the assembly was estimated to be 12 and 10 *μ*m, respectively. The coating on the assembly alone was considered a single layer, while it had two layers on the assembly and keeper.

In contrast to the report by Ahmad et al. [8], the thickness of one and two layers of coating material in this study did not produce a significant difference in reducing magnetic attraction. The differences in results were attributed to Ahmad et al. testing the density of magnetic flux, while this study examined attraction force. The scarcity of literature exploring the effect of PTFE coating on DMA led to difficulty in comparing the present results obtained.

The magnetic attraction force in group C PTFE (Table 1) has a lower value than in other groups, which might be due to the superiority of cohesive over adhesive properties of PTFE despite the lack of supporting evidence. Consequently, magnetic attraction force in group B PTFE with thicker coating material generated a stronger attraction force compared to group C PTFE.

There is a correlation between the retention of the overdenture and the attraction force of magnetic attachment. The magnetic attraction force required to provide retention in overdentures is approximately 400–600 gf [14]. The magnetic attraction force on the coated DMA in the present study exceeded 500 gf. This suggests the attraction force to be sufficient for overdenture retention despite the value being lower than the amount generated in the uncoated DMA.

The results showed that DMA coating could be applied on both the assembly and keeper surface. Furthermore, the assembly needs adequate protection because this part contains NdFeB magnet. The keeper lacks a magnet but is cemented at the tooth root or implant so that there is persistent contact with the oral cavity environment. The oral environment is known to be corrosive; therefore, magnets need to be protected.

The study by Guttal et al. [9] indicated that NdFeB magnets encased with 0.7 mm solid PTFE exhibit greater resistance to corrosion than those without casing. Varnish has been proven to prevent glass ionomer restorations from contamination of moisture [10, 11] and acidic fluids [12]. By applying varnish, it is expected that the magnet can be shielded from fluid exposure, consequently leading to corrosion prevention. Corrosion needs to be prevented because it is recognized as the most significant factor contributing to the decrease in magnetic attraction force [17]. The limitation of this study was the minimal sample size, and this was due to the difficulty of obtaining samples during the pandemic season. Further investigation is required to thoroughly examine the corrosion resistance, durability, and mechanical properties of PTFE and varnish as magnetic coating material.

## 5. Conclusion

In conclusion, the results showed that the addition of PTFE and varnish on the DMA surface tended to reduce magnetic attraction force, but the thickness of these two coating materials would have no significant influence. The coating material could be added to both assembly and keeper surfaces.

## Figures and Tables

**Table 1:** Thickness of the coating material and the magnetic attraction force.

Coating material	Group	Mean of the coating thickness (*µ*m)	Attraction force (gf)	Mean of the attraction force (gf)	Std.dev
Control	Uncoated (*A*)	—	601.16	613.63	24.55
			597.81		
			641.91		

PTFE	Assembly and keeper (*B*)	25	571.25	558.02	21.98
			570.16		
			532.64		
	Assembly (*C*)	12	532.80	534.18	11.75
			523.18		
			546.55		

Varnish	Assembly and keeper (*B*)	25	558.19	558.98	41.16
			600.52		
			518.22		
	Assembly (*C*)	10	557.15	579.61	33.20
			617.75		
			563.94		

**Table 2:** ANOVA test results for analyzed data.

Source	SS	df	MS	F	*p*-Value
Treatment	10,613.63	4	2,653.41	3.30	0.0572
Error	8,040.24	10	804.02	—	—
Total	18,653.88	14	—	—	—

## Data Availability

All data have been listed in the article.

## References

[1] Patil P. G., Nimbalkar S., Seow L. L., Kweh T. J. (2021). Unsplinted attachment systems and peri-implant outcomes in two implant retained mandibular overdentures: a systematic review of randomized controlled trials. *The Journal of Contemporary Dental Practice*.

[2] Yiu E. Y. L., Fang D. T. S., Chu F. C. S., Chow T. W. (2004). Corrosion resistance of iron–platinum magnets. *Journal of Dentistry*.

[3] Akin H., Ozdemir A. K. (2013). Effect of corrosive environments and thermocycling on the attractive force of four types of dental magnetic attachments. *Journal of Dental Sciences*.

[4] Kusumadewi A. N., Damayanti L., Rukiah R. (2021). The effect of acid solution on dental magnetic attachment. *Materials Science Forum*.

[5] Calabrese L., Caprì A., Fabiano F., Bonaccorsi L., Borsellino C., Proverbio E. (2015). Corrosion behaviour of a silane protective coating for NdFeB magnets in dentistry. *International Journal of Corrosion*.

[6] Boeckler A. F., Ehring C., Morton D., Geis-Gerstorfer J., Setz J. M. (2009). Corrosion of dental magnet attachments for removable prostheses on teeth and implants. *Journal of Prosthodontics*.

[7] Radulovic L. L., Wojcinski Z. W. (2014). PTFE (Polytetrafluoroethylene; Teflon®). *Encyclopedia of Toxicology*.

[8] Ahmad K. A., Drummond J. L., Graber T., BeGole E. (2006). Magnetic strength and corrosion of rare earth magnets. *American Journal of Orthodontics and Dentofacial Orthopedics*.

[9] Nadiger R. K., Guttal S. S. (2017). Corrosion resistance of indigenously fabricated dental magnets for application in prosthodontics. *International Journal of Prosthodontics and Restorative Dentistry*.

[10] Sukumaran V. G., Mensudar R. (2015). To evaluate the effect of surface coating on three different types glass ionomer restorations. *Biomedical and Pharmacology Journal*.

[11] Brzovic-Rajic V., Miletic I., Gurgan S., Peros K., Verzak Z., Ivanisevic-Malcic A. (2018). Fluoride release from glass ionomer with nano filled coat and varnish. *Acta Stomatologica Croatica*.

[12] Tyagi S., Thomas A. M., Sinnappah-Kang N. D. (2020). A comparative evaluation of resin and varnish-based surface protective agents on glass ionomer cement: a spectrophotometric analysis. *Biomaterial Investigations in Dentistry*.

[13] Kang S.-Y., Yu J.-M., Kim H.-S., Park K.-S., Lee S.-Y. (2019). A study on performance evaluation of magnetic dental attachments. *Journal of Magnetics*.

[14] Lee E., Shin S.-Y. (2017). The influence of the number and the type of magnetic attachment on the retention of mandibular mini implant overdenture. *The Journal of Advanced Prosthodontics*.

[15] Akin H., Coskun M. E., Akin E. G., Ozdemir A. K. (2011). Evaluation of the attractive force of different types of new-generation magnetic attachment systems. *The Journal of Prosthetic Dentistry*.

[16] Chung K.-H., Whiting D., Kronstrom M., Chan D., Wataha J. (2011). Retentive characteristics of overdenture attachments during repeated dislodging and cyclic loading. *The International Journal of Prosthodontics*.

[17] Kusumadewi A. N., Damayanti L., Rukiah, Risdiana (2022). Factors affecting the attractive force of dental magnetic attachment: a literature review for guiding dentists in clinical application. *International Journal of Dentistry*.

